# Supplementary material for: Leukemia risk factor ARID5B coordinates HDAC-mediated transcriptional repression
Source: Nucleic Acids Res. 2026 Jun 22;54(12):gkag628. doi: 10.1093/nar/gkag628 (PMC13284713; doi:10.1093/nar/gkag628)
Supplement: gkag628_Supplemental_Files [file gkag628_supplemental_files.zip › NAR_revision_Supplementary_Figures.pdf]

Supplementary Figures S1 to S8

Leukemia risk factor ARID5B Coordinates HDAC-Mediated Transcriptional Repression

Ana P. Kutschat[1,2], Fabian Frommelt[2,5], Brianda L. Santini[2,5], Sophie Müller[1,2], Paul Batty[1,2], Animesh Awasthi[2,3], Gerlinde Karbon[1,2], Giulio Superti-Furga[2,4], Davide Seruggia[1,2,*]

[1] St. Anna Children's Cancer Research Institute (CCRI), Vienna, Austria
[2] CeMM Research Center for Molecular Medicine of the Austrian Academy of Sciences, Vienna, Austria
[3] Medical University of Vienna, Institute of Artificial Intelligence, Center for Medical Data Science, Vienna, Austria
[4] Center for Physiology and Pharmacology, Medical University of Vienna, Vienna, Austria
[5] These authors contributed equally
* Correspondence: davide.seruggia@ccri.at

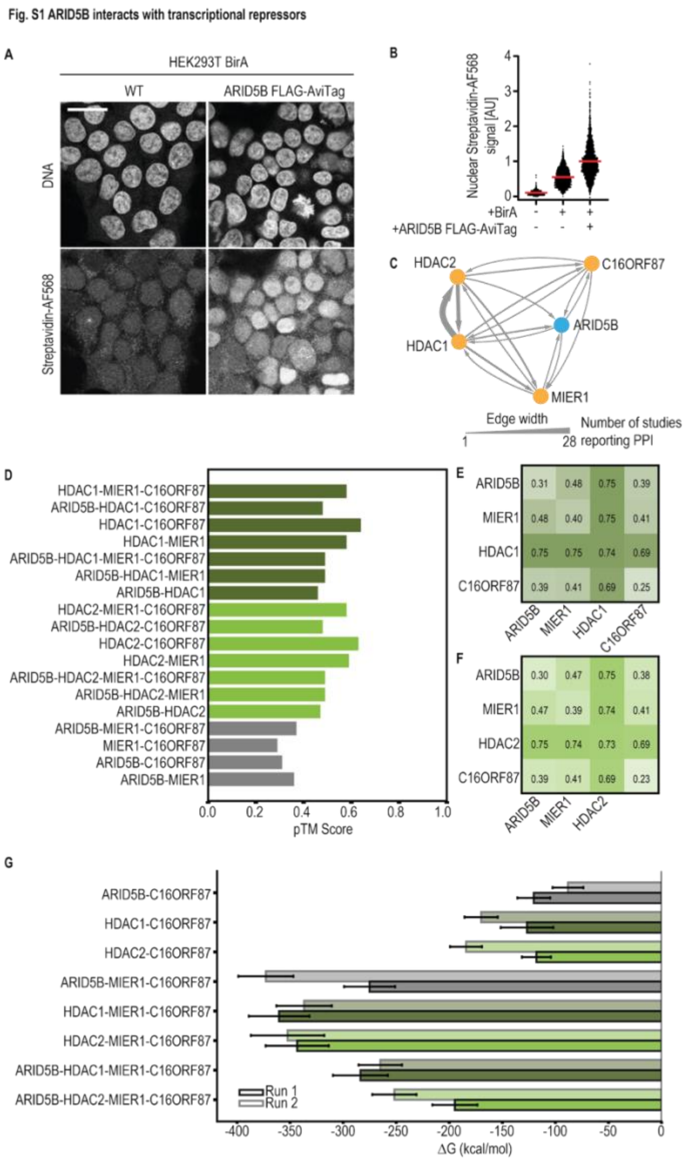

Figure S1: ARID5B interacts with transcriptional repressors

a. Representative immunofluorescence images of HEK293T cells stably expressing the E. Coli BirA biotin ligase, with or without expression of a murine ARID5B FLAG-AVI-tag cDNA construct. Cells were fixed for immunofluorescence and biotin deposition was assessed using Streptavidin conjugated to Alexa Fluor 568. DNA was stained with Hoechst 33342. Scale bar: 20 µm. **b.** Quantification of nuclear Streptavidin AF568 fluorescence by immunofluorescence for cells in **(a)** Wild type HEK293T cells were also used as a negative control to measure background staining. Dots represent the mean nuclear Streptavidin AF568 fluorescence of individual cells; red bars indicate the mean. Significance was tested using a two-tailed Mann–Whitney U-test. Number of cells analyzed (merge of *n* = 2 biological replicates): WT (*n* = 1255), BirA overexpression (*n* = 2032), BirA + ARID5B overexpression (*n* = 2672). **c.** Directional PPI-network of ARID5B co-repressor complex subunits obtained from publicly available interaction proteomics datasets. Edge arrows indicate the direction of interaction as reported in the database, while edge thickness corresponds to the number of associated publications. **d.** Predicted template modelling (pTM) scores from AlphaFold3 models for all predicted complex combinations. **e., f.** Chain-pair ipTM score matrices for AlphaFold3-predicted ARID5B-MIER1-C16ORF87 complexes with HDAC1 **(e)** or HDAC2 **(f)**. **g.** Total binding free energy of C16ORF87 in protein complexes with ARID5B, HDAC1, HDAC2 and MIER1. The mean total binding free energy contribution of C16ORF87 to each complex masking the other subunits obtained through MM/GBSA across the trajectory. Error bars indicate standard deviation across the MD trajectories. The x-axis shows the ΔG, and the y-axis specific protein complexes, sorted from binary interactions of C16ORF87 to tetrameric complexes. For the analysis, two independent molecular dynamic simulation runs were conducted, Run 1 and Run 2, respectively.

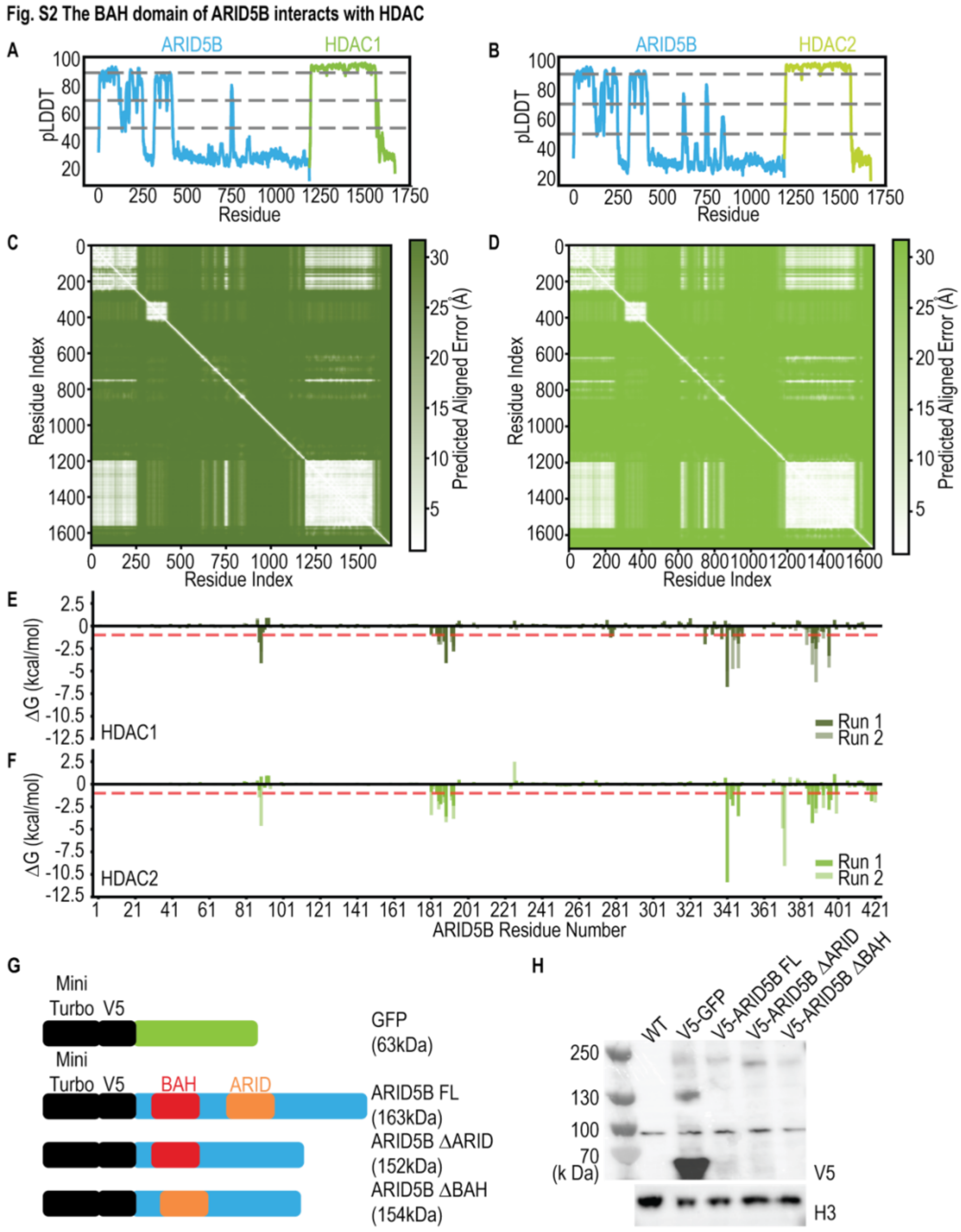

Figure S2: The BAH domain of ARID5B interacts with HDAC

a., b. Per-residue predicted local distance difference test (pLDDT) scores for AlphaFold3-predicted ARID5B-HDAC1 **(a)** and ARID5B-HDAC2 **(b)** complexes. **c., d.** Predicted aligned error (PAE) heatmaps for ARID5B-HDAC1 **(c)** and ARID5B-HDAC2 **(d)** complexes. **e-f.** Per-residue binding free energy decomposition analysis of ARID5B interacting with HDAC1 (**e**) and HDAC2 (**f**). The top shows the binding free energy contributions (ΔG, kcal/mol) for each ARID5B residue interacting with HDAC1 (**e**), while the bottom shows the interaction with HDAC2 (**f**). The x-axis shows the ARID5B residue index, and the y-axis the ΔG. The read dashed line indicates a threshold of -1.0 kcal/mol. For the analysis, two independent molecular dynamic runs were conducted, Run 1 and Run 2, respectively. **g.** Schematic overview of the constructs used for co-immunoprecipitation experiments. GFP, full-length (FL) ARID5B and ARID or BAH domain deletion mutants (ΔARID or ΔBAH, respectively) containing a N-terminus MiniTurbo and V5 Tag were overexpressed in HAP1 cells expressing Cas9. **h.** Assessment of overexpression constructs for co-immunoprecipitation. Western Blot for V5 of protein lysates of HAP1 Cas9 and HAP1 Cas9 overexpressing V5-tagged GFP, full-length (FL) ARID5B and ARID or BAH domain deletion mutants (ΔARID or ΔBAH, respectively). Expected protein sizes are 63 kDa, 163 kDa, 152 kDa and 154 kDa for GFP, ARID5B FL, ΔARID and ΔBAH, respectively. H3 was used as a loading control.

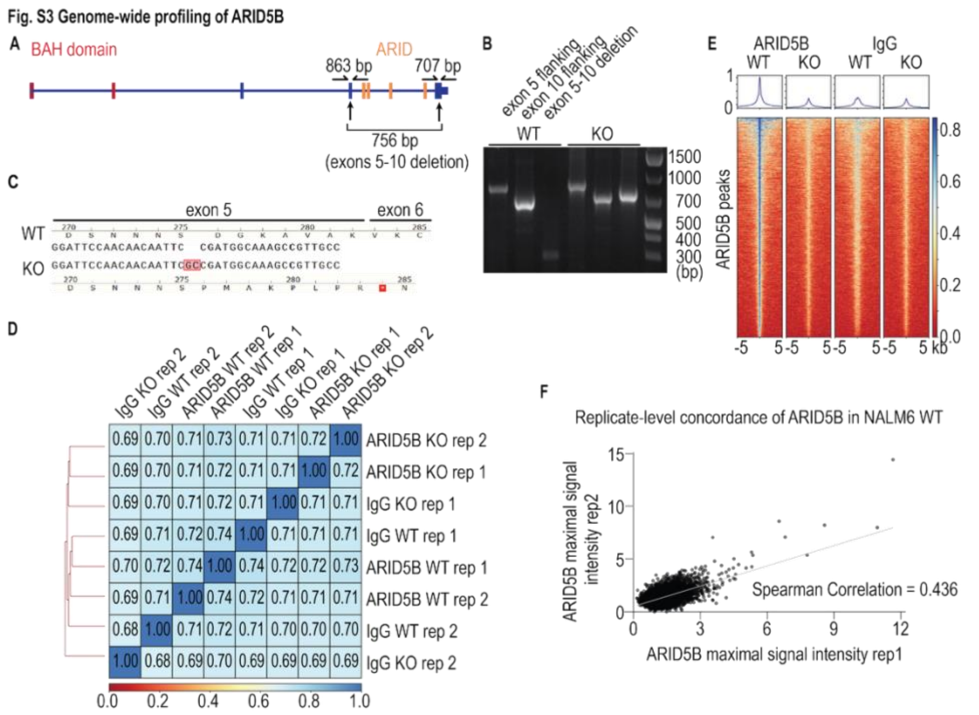

Figure S3: Genome-wide profiling of ARID5B

a. Scheme depicting the strategy for *ARID5B* KO. NALM6 WT cells were electroporated with RNPs with sgRNAs targeting *ARID5B* exons 5 and 10. Primers flanking the targeted regions were used to assess deletion of the exon 5-10 region as well as indels and point mutations. **b.** Genotyping of NALM6 WT and ARID5B KO cells. As expected, in NALM6 WT cells, PCR products are only observed for regions flanking the targeted *ARID5B* loci. ARID5B KO cells appear to be compound heterozygous, with one frameshift allele retaining exons 5 and 10 and the other allele presenting a deletion of the *ARID5B* exon 5-10 segment. **c.** Sanger sequencing of the targeted exon 5 in ARID5B KO cells reveals a 2 bp insertion, leading to a premature stop codon. **d.** Replicate-level spearman correlation matrix of ARID5B and IgG CUT&RUN signal in NALM6 WT and ARID5B KO cells across the entire human genome. The genome wide coverage (bigwig files) of each replicate was used for the analysis. **e.** Aggregate plots and heatmaps of ARID5B and IgG in NALM6 WT and ARID5B KO cells centered on the previously determined 4,200 ARID5B peaks. The coverage of two merged biological replicates per ARID5B and IgG per genotype was used for the analysis. **f.** Spearman correlation of ARID5B signal intensity across replicates on the identified 4,200 ARID5B peaks. The average ARID5B signal intensity for each region was calculated within +/-5kb bins using *compute matrix* from deeptools. The median signal intensity was plotted and used for the calculation of the spearman correlation coefficient.

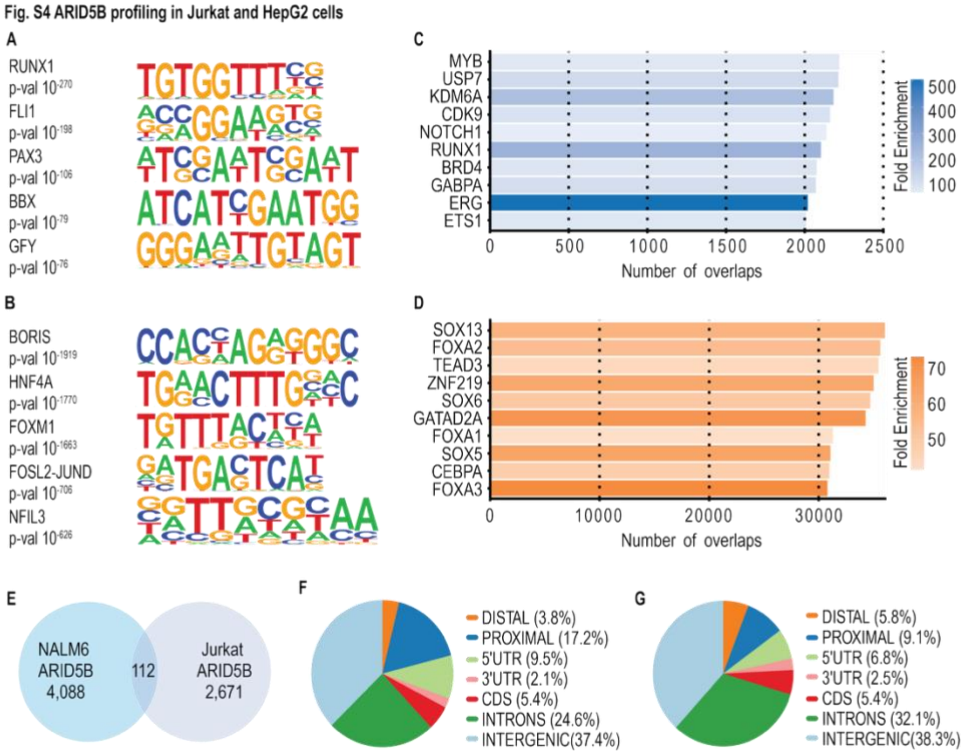

Figure S4: ARID5B profiling in Jurkat and HepG2 cells

a-b. Five most significant HOMER *de novo* motifs enriched on ARID5B peaks in Jurkat (**a**) and HepG2 (**b**) cells**. c-d.** Enrichment analysis using ChIP Atlas of publicly available TFs or co-factors co-localizing with ARID5B in Jurkat (**c**) and HepG2 (**d**)**.** The analysis in Jurkat and HepG2 was restricted to factors probed in blood and liver cells by setting the "Cell Class" filter to "Blood" and to "Liver", respectively. The bar plot depicts the top factors and their overlap with ARID5B occupied regions. Only data of endogenous, non-tagged, ChIP-seq in "blood" (depicted are overlaps of ChIP-seq peaks in Jurkat, MOLT-4, HPB-ALL, CUTLL1 and CTV-1) and "liver" (depicted are overlaps of ChIP-seq peaks in HepG2) cells with an FDR ≤ to 0.05 is shown. **e.** Venn Diagram of ARID5B peaks in NALM6 and Jurkat cells. **f-g.** Genomic distribution of ARID5B peaks called in Jurkat (**f**) and HepG2 (**g**) cells.

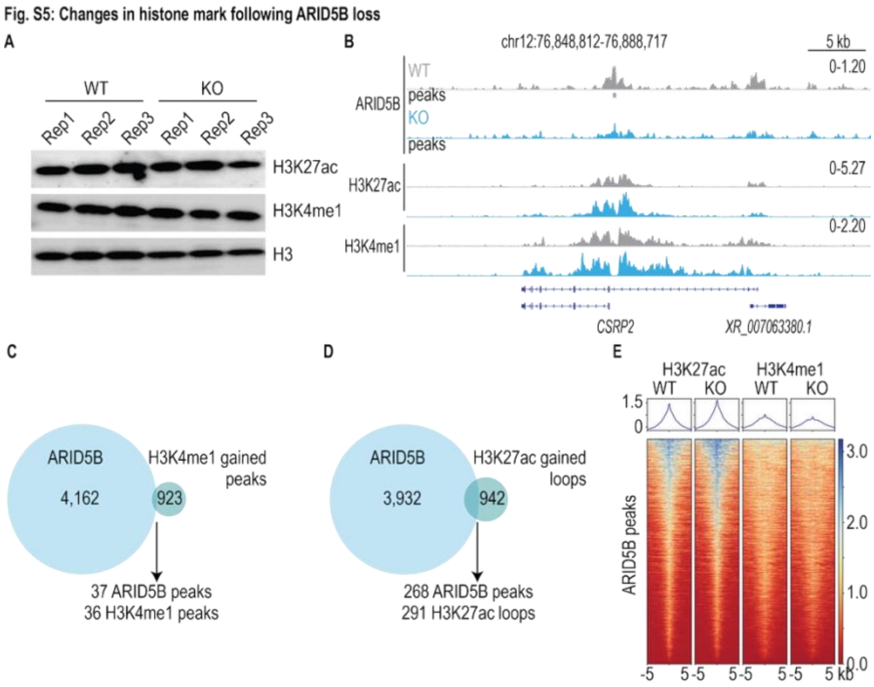

Figure S5: Changes in histone mark following ARID5B loss

a. H3K4me1 and H3K27ac protein levels in NALM6 WT and ARID5B KO cells. The Western Blot was run using biological triplicates. H3 serves as a protein loading control. **b** Genome-wide coverage of ARID5B, H3K27ac and H3K4me1 in NALM6 WT and NALM6 ARID5B KO cells at the *CSRP2* locus. The coverage of two merged biological replicates per histone mark and ARID5B per genotype was used for the analysis. **c.** Venn Diagram of the 4,200 ARID5B peaks in NALM6 with H3K4me1 peaks gained upon ARID5B loss. **d.** Venn Diagram of the 4,200 ARID5B peaks in NALM6 with H3K27ac loops gained upon ARID5B loss. Gained loops were obtained by intersecting H3K27ac gained peaks upon ARID5B KO with H3K27ac loops from H3K27ac HiChIP in NALM6 cells. **e.** Aggregate plots and heatmaps of H3K27ac and H3K4me1 in NALM6 WT and ARID5B KO cells centered on the previously determined 4,200 ARID5B peaks. The coverage of two merged biological replicates per histone mark was used for the analysis.

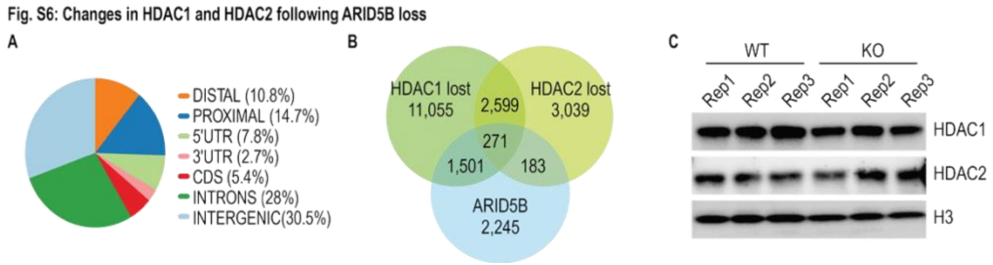

Figure S6: Changes in HDAC1 and HDAC2 following ARID5B loss

a. Genomic distribution of 2,872 ARID5B peaks bound by HDAC1 and HDAC2, as identified in Fig. 3A. **b.** Venn Diagram of ARID5B peaks in NALM6 cells losing HDAC1 and/or HDAC2 binding upon ARID5B loss. **c.** HDAC1 and HDAC2 protein levels in NALM6 WT and ARID5B KO cells. The Western Blot was run using biological triplicates. H3 serves as a protein loading control and was depicted previously in Fig. S5A.

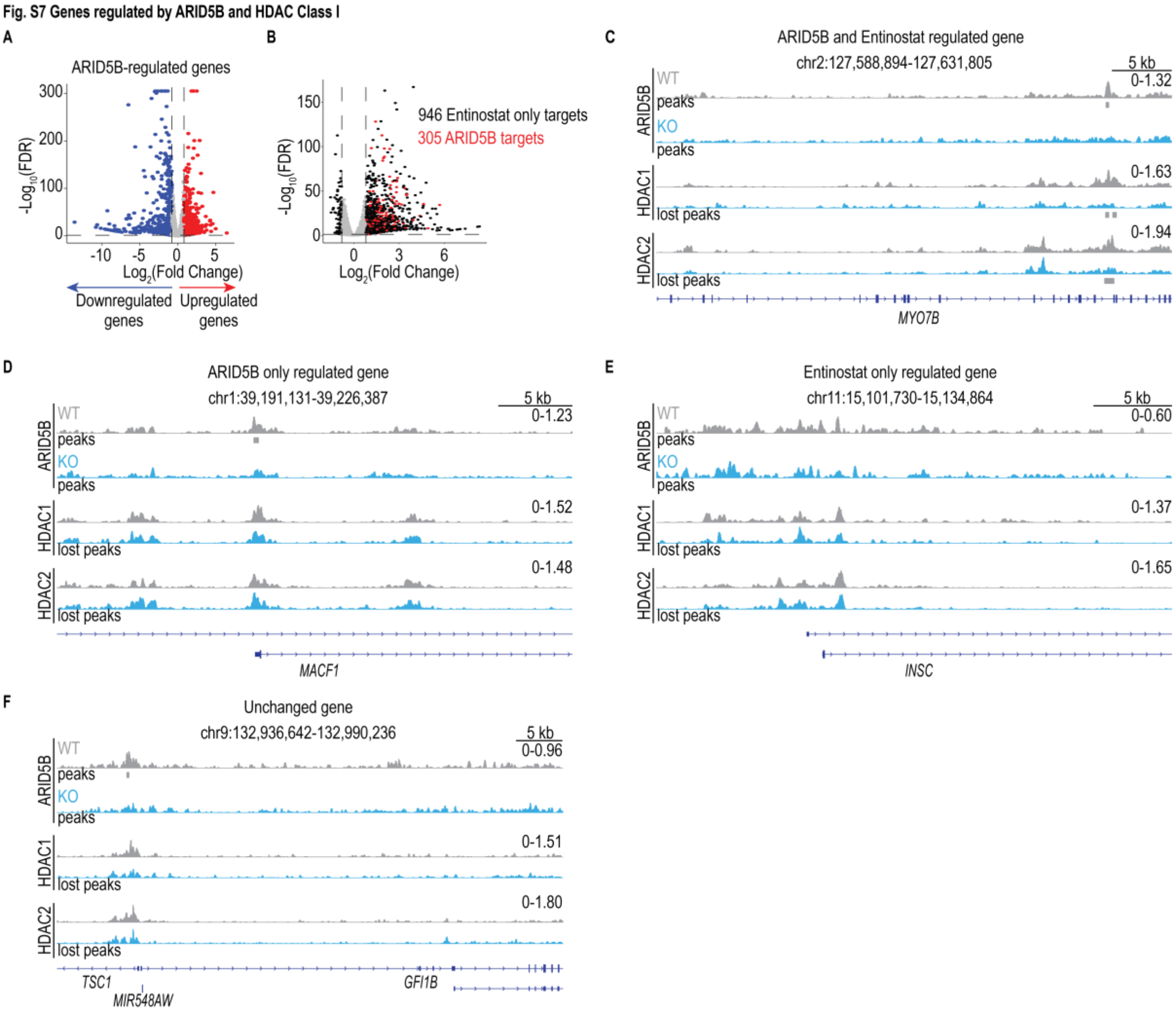

Figure S7: Genes regulated by ARID5B and HDAC Class I

a. Volcano plot of genes regulated ARID5B KO in NALM6 cells. Highlighted in red and blue are up- and downregulated genes, respectively. Dotted lines indicate a log2FC threshold of larger or equal to 0.8 and a FDR threshold of smaller or equal to 0.05. The following thresholds were used for up- (log2FC ≥ 0.8, padj ≤ 0.05, cpm (KO) ≥ 30) and downregulated (log2FC ≤ -0.8, padj ≤ 0.05, cpm (WT) ≥ 30) genes. Biological triplicates per genotype and per treatment were used. **b.** Volcano plot of genes regulated upon Entinostat treatment in NALM6 WT cells. Highlighted in red are Entinostat-responsive genes that are also upregulated in ARID5B KO cells. Dotted lines indicate a log2FC threshold of larger or equal to 0.8 and a FDR threshold of smaller or equal to 0.05. The following thresholds were used for up (log2FC ≥ 0.8, padj ≤ 0.05, cpm (KO/Entinostat) ≥ 30) and downregulated (log2FC ≤ -0.8, padj ≤ 0.05, cpm (WT/DMSO) ≥ 30) genes. NALM6 WT cells were treated with vehicle or 500 nM Entinostat for 24 h prior to mRNAseq experiment. Biological triplicates per genotype and per treatment were used. **c-f.** Genome-wide coverage of ARID5B, HDAC1 and HDAC2 in WT and ARID5B KO NALM6 cells at genes upregulated by ARID5B and Entinostat (*MYO7B*) (**c**), by ARID5B only (*MACF1*) (**d**), by Entinostat only (*INSC*) (**e**) and at unchanged genes *(TSC1 GFI1B*) (**f**). The coverage of two merged biological replicates of HDAC1, HDAC2 or ARID5B per genotype is shown.

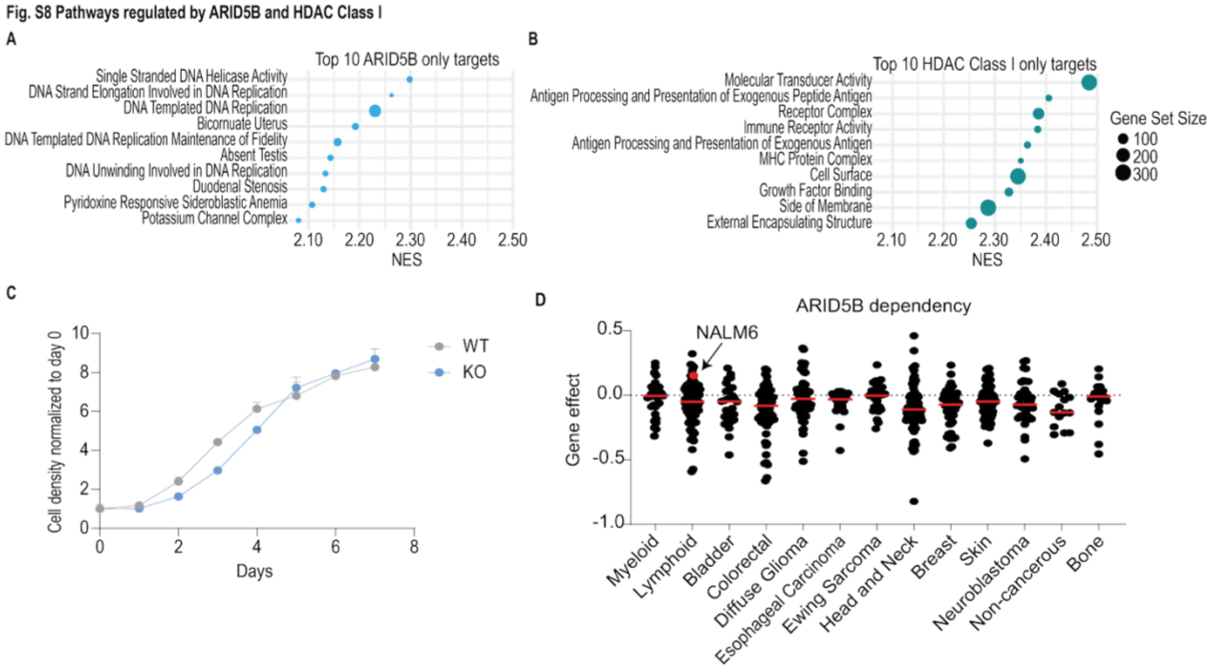

Figure S8: Pathways regulated by ARID5B and HDAC Class I

a. Top 10 Gene Ontology terms enriched in gene set enrichment analysis (GSEA – C2 collection) of ARID5B KO NALM6 cells only. Depicted are the normalized enrichment scores (NES) and the number of genes contained in each gene ontology term. GSEA was run comparing NALM6 WT with ARID5B KO cells. Terms with an FDR smaller or equal to 0.25 were considered statistically significant. **b.** Top 10 Gene Ontology terms enriched in gene set enrichment analysis (GSEA - C2 collection) of Entinostat treated NALM6 cells only. Depicted are the NES and the number of genes contained in each gene ontology term. GSEA was run comparing vehicle-treated with Entinostat-treated cells. Terms with an FDR smaller or equal to 0.25 were considered statistically significant. NALM6 WT cells were treated with vehicle or 500 nM Entinostat for 24 h prior to mRNAseq experiment. **c.** Proliferation assay of NALM6 WT and ARID5B KO cells. Biological duplicates were seeded and counted daily for 7 days. Cell density is normalized to day 0, day of seeding. **d.** DepMap data (25Q3 + Score Chronos) on ARID5B dependency on cell lines of various cancer types, including NALM6 (highlighted in red).
